# Latent profiles of volume management behaviors and their relationship with symptom distress in patients with chronic heart failure

**DOI:** 10.3389/fcvm.2025.1698319

**Published:** 2026-01-07

**Authors:** Weiwei Liu, Yuzhong Gu, Qingqing Yang, Yi Lu

**Affiliations:** 1 Department of Cardiology, The Fourth People’s Hospital of Nantong, Nantong, Jiangsu, China; 2 Department of Cardiology, The Sixth People’s Hospital of Nantong, Nantong, Jiangsu, China

**Keywords:** chronic heart failure, latent profile analysis, older, symptom distress, volume management behavior

## Abstract

**Objective:**

To explore the latent categories of volume management behaviors in patients with chronic heart failure (CHF) and analyze their relationship with symptom distress.

**Methods:**

This cross-sectional study utilized a convenience sampling method to select 552 CHF patients from the cardiology departments of Nantong Sixth People's Hospital and Nantong Fourth People's Hospital. Volume management behaviors were assessed using the Volume Management Behavior Scale, and symptom distress was evaluated using the Symptom Distress Questionnaire (SDQ), which measures the severity of eight core symptoms. Latent Profile Analysis (LPA) was employed to identify behavioral categories. Multivariate Analysis of Variance (MANOVA) and multiple linear regression were used to analyze differences in symptom distress across behavioral categories and to examine the independent predictive effect of behavioral classification on symptom distress.

**Results:**

The volume management behaviors of CHF patients were classified into three latent categories: active management type (43.1%), selective adherence type (27.7%), and passive dependence type (29.2%). Symptom distress scores showed a significant increasing trend across the three categories (active type: 10.5 ± 3.8; selective type: 13.2 ± 4.1; passive type: 16.3 ± 5.2, *P* < 0.001). After controlling for confounding factors such as age, gender, and NYHA classification, behavioral categories independently explained 41% of the total variance in symptom distress (adjusted *R*^2^ = 0.41, *F* = 32.17, *P* < 0.001), with the passive dependence type demonstrating the strongest predictive effect (*β* = 5.82, 95% CI: 4.21–7.43).

**Conclusion:**

CHF patients exhibit three distinct clinical patterns of volume management behaviors, with the passive dependence type associated with the highest symptom burden. Behavioral category is a significant predictor of symptom distress. These findings provide an empirical basis for developing precise intervention strategies tailored to different behavioral phenotypes.

## 1 Introduction

Chronic heart failure (CHF) represents the end stage of various cardiovascular diseases and is characterized by high incidence rates, high readmission rates, and a substantial disease burden (1). Volume overload is a core pathophysiological mechanism in CHF, serving as one of the primary causes of acute exacerbations and recurrent hospitalizations (2, 3), and a direct driver of severe symptom distress in patients (4). Multidimensional symptom distress triggered by volume overload—such as dyspnea, fluid retention (edema, rapid weight gain), and extreme fatigue—constitutes the core of the illness experience for CHF patients (5, 6). These symptoms not only severely restrict patients' daily activities but also evoke anxiety, fear, and helplessness, significantly undermining their quality of life and self-management confidence (7, 8). Studies indicate that high levels of symptom distress are an independent predictor of readmission and poor prognosis (5). Therefore, effectively alleviating symptom distress is a central goal of CHF management. Achieving this goal hinges on controlling volume overload at its source through effective volume management behaviors. These include sodium and fluid restriction, regular monitoring of weight and symptoms, adherence to medication (particularly diuretics), and timely seeking of medical care (9, 10). These behaviors form a critical bridge between clinical guidelines and patients' symptom experiences. The Volume Management Behavior Scale developed by Ning Li (11) encompasses multidimensional behaviors ranging from monitoring and evaluation to maintenance and emergency response, providing a scientific tool for systematically assessing these behavioral patterns.

However, existing research predominantly relies on total scale scores or unidimensional compliance indicators to simplistically dichotomize patient behavior into “high/low adherence” (12, 13). This approach fails to explain common clinical paradoxes, such as why some “compliant” patients still endure severe symptom distress. It overlooks the significant individual heterogeneity in patients' complex combinations of volume management behaviors—for example, being proactive in monitoring but delayed in response, or having adequate understanding but insufficient confidence. This heterogeneity may be a key, yet long-neglected, reason for the vast differences in symptom experiences among patients.

Latent Profile Analysis (LPA), as a person-centered statistical method, can identify latent behavioral subpopulations within the patient group based on multiple observed behavioral indicators (14). This offers a novel perspective for systematically revealing the correspondence between different behavioral patterns and specific levels of symptom distress. Currently, few empirical studies have applied LPA to explore the potential categories of volume management behaviors in CHF patients. More importantly, how these distinct latent behavioral patterns lead to systematic differences in patients' symptom distress levels remains insufficiently elucidated. Therefore, this study aims to use LPA to explore the potential categories (profiles) of volume management behaviors among CHF patients and to clarify the association patterns between these behavioral profiles and patients' levels of symptom distress. This research seeks to answer: Do certain high-risk behavioral profiles exist, characterized not only by “poor compliance” but also by “imbalanced behavioral combinations,” which cause these subpopulations to bear a disproportionately severe symptom burden? The findings are expected to provide an empirical basis for developing refined, targeted volume management intervention strategies aimed directly at alleviating symptom distress.

## 2 Subjects and methods

### 2.1 Subjects

This cross-sectional study utilized a convenience sampling method to select patients with chronic heart failure (CHF) who were admitted to the Department of Cardiology at Nantong Sixth People's Hospital and Nantong Fourth People's Hospital between August 2024 and April 2025. Inclusion criteria: (1) Age ≥ 18 years; (2) Meeting the diagnostic criteria for chronic heart failure as defined in the Chinese Guidelines for the Diagnosis and Treatment of Heart Failure 2024 (9), with the disease in a relatively stable phase; (3) New York Heart Association (NYHA) functional class I–IV; (4) Clear consciousness, no history of mental illness, able to understand questionnaire content, and no communication barriers; (5) Possessing basic literacy skills or able to complete the questionnaire independently with researcher assistance. Exclusion criteria: (1) Acute heart failure or complicated by cardiogenic shock; (2) Comorbidities such as malignant tumors, severe immune system diseases, or end-stage liver/kidney failure (eGFR <30 mL/min/1.73 m^2^); (3) Severe visual or hearing impairments preventing cooperation with questionnaire surveys; (4) Moderate to severe cognitive impairment (MMSE score ≤ 18) (15) or mental disorders such as major depression or schizophrenia.The study protocol was reviewed and approved by the Ethics Committee of Nantong Sixth People's Hospital (Approval No. NTLYLL2024063). All patients provided written informed consent. Based on LPA requirements (16), each latent class should have a sample size >5%–10% of the total sample, with an absolute number >50. Assuming the smallest expected class proportion is 10%, the total sample size must satisfy: *N* × 10% ≥ 50, hence *N* ≥ 500. Ultimately, 559 patients were included in this study.

### 2.2 Survey instruments

#### 2.2.1 General information questionnaire

Data were collected using a self-designed questionnaire. General demographic data were obtained through patient interviews. Disease-related clinical parameters, including NYHA functional class, left ventricular ejection fraction (LVEF), brain natriuretic peptide (BNP) levels, number of comorbidities, and diuretic use, were assessed by the research nurses based on a review of the patients' medical records.

#### 2.2.2 Symptom distress assessment

Symptom distress was evaluated using the Heart Failure-Specific Symptom Distress Questionnaire (SDQ) developed by Shiao-Pei Wang (17), which assesses the subjective discomfort experienced by patients due to the disease and its treatment. The scale covers eight core symptoms of heart failure: dyspnea, edema, fatigue, palpitations, chest tightness, loss of appetite, dizziness, and weight gain. Each symptom is rated on a 4-point scale based on the level of distress: 0 = no distress to 3 = extreme distress. The total score ranges from 0 to 24, with higher scores indicating more severe symptom distress. In this study, the Cronbach's α of the scale was 0.82, indicating good reliability.

#### 2.2.3 Volume management behavior scale

The Volume Management Behavior Scale for patients with chronic heart failure, compiled by Ning Li in 2024 (11), was used. The scale consists of 27 items divided into four dimensions: self-care monitoring (12 items), self-care maintenance (5 items), self-care management (6 items), and self-care confidence (4 items). All items are scored on a 5-point Likert scale, and the total score ranges from 27 to 135. Higher scores indicate better volume management behavior. In this study, the Cronbach's α of the scale was 0.82, indicating good reliability.

### 2.3 Survey methods and quality control

Researchers provided the Informed Consent Form to patients within 24 h after discharge, thoroughly explaining the study purpose, content, and confidentiality principles. Data collection commenced only after written consent was obtained. This study followed a standardized survey protocol implemented simultaneously at Nantong Sixth People's Hospital and Nantong Fourth People's Hospital. Prior to data collection, five uniformly trained cardiac research nurses (two from each hospital plus one coordinator) carried out the following standardized procedures: within 24 h of discharge, they explained the study objectives, confidentiality principles, and questionnaire content to patients, and initiated the assessment only after written informed consent was secured; face-to-face interviews were conducted using unified instructions, and when patients had questions about specific items—such as technical terms related to volume management—standardized neutral explanations were provided; questionnaires were checked on-site for completeness immediately after administration, with an allowable item missing rate of less than 5%; additionally, 10% of the questionnaires from both hospitals were cross-verified weekly, and those exhibiting patterned responses or logical inconsistencies were excluded. A total of 559 eligible CHF patients were initially enrolled. After excluding 7 invalid questionnaires (3 due to patterned responses and 4 due to critical missing data), the final valid sample size was 552, resulting in an effective response rate of 98.7%.

### 2.4 Statistical analysis

LPA was performed using Mplus 8.3 software, with the raw scores of the 27 items from the Volume Management Behavior Scale serving as manifest variables. Competing models with 1–5 latent classes were fitted. The optimal model was selected based on the following criteria: The optimal model was selected based on the following criteria: smallest AIC, BIC, and aBIC values; an entropy value >0.8 was employed to assess the precision of individual classification into latent profiles; and statistically significant results (*P* < 0.05) for both the Lo-Mendell-Rubin Test (LMRT) and the Bootstrap Likelihood Ratio Test (BLRT), indicating that the addition of an extra latent class significantly improved model fit, while also considering clinical interpretability. All other analyses were conducted using SPSS 26.0. Descriptive statistics were presented as mean ± standard deviation $\left(\bar{x}\pm s\right)$ for normally distributed continuous variables, median (interquartile range) [M (P25, P75)] for non-normally distributed variables, and frequency (percentage) [*n* (%)] for categorical variables. Group comparisons were performed using ANOVA or the Kruskal–Wallis *H* test for continuous variables depending on homogeneity of variance, and the chi-square test or Fisher's exact test for categorical variables. One-way ANOVA was used to examine overall differences in SDQ scores across different latent behavior profiles. If statistically significant differences were observed (*P* < 0.05), *post-hoc* pairwise comparisons were conducted using the Least Significant Difference (LSD) method due to the exploratory context. The relationship between behavior profiles and SDQ scores was further validated using multiple linear regression, adjusting for covariates such as age, gender, NYHA class, and LVEF. All analyses employed two-sided tests, with *P* < 0.05 considered statistically significant.

## 3 Results

### 3.1 General characteristics of the study participants

The mean age of the CHF patients in this study was 68.5 ± 9.2 years. The majority were male (56.5%) and had moderate to severe heart failure (NYHA class II–III, accounting for 90%). Detailed characteristics are presented in Table 1.

**Table 1:** General characteristics of the study participants (*N* = 552).

Characteristic	Statistical value	Percentage (%)
Age (years)	68.5 ± 9.2	–
Gender		
Male	312	56.5%
Female	240	43.5%
Education Level		
Primary school or below	197	35.7%
Junior high school	215	39.0%
High school/Technical school	98	17.8%
College or above	42	7.6%
Marital Status		
Married/Cohabiting	486	88.0%
Widowed/Divorced	66	12.0%
NYHA Functional Class		
Class II	254	46.0%
Class III	243	44.0%
Class IV	55	10.0%
LVEF(%)	40.2 ± 8.9	–
NT-proBNP (pg/mL)	6,580 (3,210, 12,500)	–
Number of Comorbidities		
0–1	138	25.0%
2	245	44.4%
≥3	169	30.6%
Diuretic Use [*n* (%)]		
Not used	64	11.6%
Loop diuretics	388	70.3%
MRA	218	39.5%
Thiazides	40	7.2%

MRA, mineralocorticoid receptor antagonist.

### 3.2 Latent profile analysis results and naming of volume management behaviors in CHF patients

LPA was conducted on the 27 items of the Volume Management Behavior Scale using Mplus 8.3 software Competing models with 1–5 latent classes were fitted, as shown in Table 2 and The results indicated that the 3-class model had lower AIC (16,238.71), BIC (16,524.78), and aBIC (16,298.55) values compared to the 1- and 2-class models (*P* < 0.05), with both the LMR test and BLRT being statistically significant (*P* < 0.001). Although the 4-class model had slightly lower AIC/BIC values, the LMR test was not significant (*P* = 0.11). The 5-class model showed a further decrease in BIC but had an Entropy value <0.8 and a non-significant LMR test (*P* = 0.072). Therefore, the 3-class model was accepted as the optimal solution.

**Table 2:** Model fit indices for latent classes of volume management behaviors in CHF patients.

Model	AIC	BIC	aBIC	Entropy	LMR (*P*)	BLR (*P*)	Class proportions
1	17,528.93	17,686.57	17,549.81	–	–	–	–
2	16,492.35	16,714.20	16,532.71	0.872	<0.001	<0.001	0.552/0.448
3	16,238.71	16,524.78	16,298.55	0.901	<0.001	<0.001	0.431/0.277/0.292
4	16,189.42	16,539.70	16,268.74	0.813	0.118	<0.001	0.218/0.304/0.192/0.286
5	16,105.83	16,520.32	16,204.63	0.794	0.072	<0.001	0.185/0.210/0.197/0.208/0.200

CHF, chronic heart failure; AIC, akaike information criterion; BIC, bayesian information criterion; aBIC, sample-adjusted bayesian information criterion; LMR, lo-mendell-rubin test; BLR, bootstrap likelihood ratio test.

**Figure 1:**
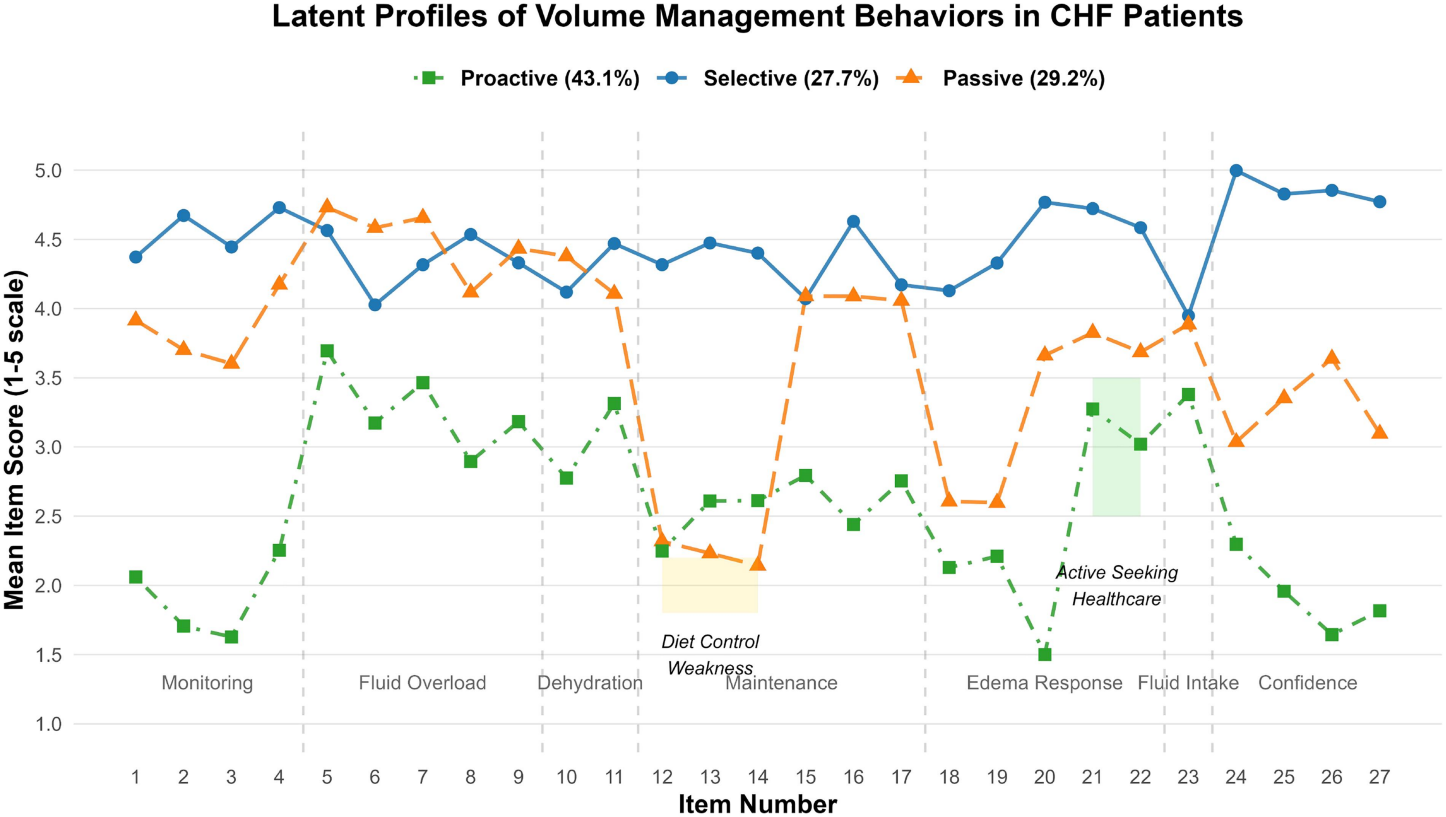
Scores of each item of volume management behavior in CHF patients.

The behavioral dimension characteristics of each latent class are shown in Figure 1. Class 1 (*n* = 238, 43.1%) scored significantly higher on the self-care confidence dimension (items 24–27) compared to other classes and was thus designated as the Active Management Type. Class 2 (*n* = 153, 27.7%) scored high on items 5–11 (symptom recognition) but low on items 12–14 (sodium restriction) and items 18–20 (edema response), and was thus designated as the Selective Adherence Type. Class 3 (*n* = 161, 29.2%) scored the lowest across all dimensions and was designated as the Passive Dependence Type.

### 3.3 Univariate analysis of latent classes in volume management among CHF patients

Univariate analysis of the latent classes in volume management revealed that age, gender, education level, NYHA functional class, LVEF, NT-proBNP, number of comorbidities, and symptom distress showed statistically significant differences (all *P* < 0.05), as shown in Table 3.

**Table 3:** Univariate analysis of patients with different latent volume management profiles (*n* = 552).

Variable	Active management type (*n* = 238)	Selective adherence type (*n* = 153)	Passive dependence type (*n* = 161)	Statistic	*P*-value
Age (years)	65.2 ± 8.7	69.8 ± 8.9	71.3 ± 9.5	*F* = 18.32	<0.001
Gender [*n* (%)]					
Male	150 (63.0%)	85 (55.6%)	77 (47.8%)		
Female	88 (37.0%)	68 (44.4%)	84 (52.2%)	*χ*^2^ = 8.76	0.012^†^
Education Level [*n* (%)]					
Junior high school or below	153 (64.3%)	118 (77.1%)	141 (87.6%)		
High school or above	85 (35.7%)	35 (22.9%)	20 (12.4%)	*χ*^2^ = 25.31	<0.001^†^
NYHA Functional Class [*n* (%)]^§^				*χ*^2^ = 32.15	<0.001^†^
Class II	140 (58.8%)	68 (44.4%)	46 (28.6%)		
Class III	86 (36.1%)	75 (49.0%)	82 (50.9%)		
Class IV	12 (5.0%)	10 (6.5%)	33 (20.5%)		
LVEF (%)	43.5 ± 7.8	39.8 ± 8.2	37.1 ± 9.3	*F* = 24.67	<0.001
NT-proBNP (pg/mL)	3,850 (2,010, 7,520)	6,850 (3,320, 11,200)	12,500 (7,450, 18,400)	*H* = 37.55	<0.001^‡^
Number of Comorbidities				*χ*^2^ = 34.28	<0.001^†^
0–1	78 (32.8%)	35 (22.9%)	25 (15.5%)		
2	110 (46.2%)	78 (51.0%)	57 (35.4%)		
≥3	50 (21.0%)	40 (26.1%)	79 (49.1%)		
Diuretic Use [*n* (%)]					
No diuretics	28 (11.8%)	18 (11.8%)	18 (11.2%)	*χ*^2^ = 0.05	0.975^†^
Loop diuretics	168 (70.6%)	108 (70.6%)	112 (69.6%)	*χ*^2^ = 0.06	0.971^†^
MRA	94 (39.5%)	64 (41.8%	60 (37.3%)	*χ*^2^ = 0.78	0.677^†^
Thiazides	20 (8.4%)	15 (9.8%)	15 (9.3%)	*χ*^2^ = 0.31	0.856^†^
Total Symptom Distress Score	10.5 ± 3.8	13.2 ± 4.1	16.3 ± 5.2	F = 72.83	<0.001

† *χ*^2^ test.

‡ Kruskal–Wallis *H* test.

§ NYHA class was analyzed as an ordinal variable using the Cochran-Armitage trend test (*Z* = 5.22, *P* < 0.001).

### 3.4 Correlation between latent classes of volume management behaviors and symptom distress dimensions in CHF patients

**Figure 2:**
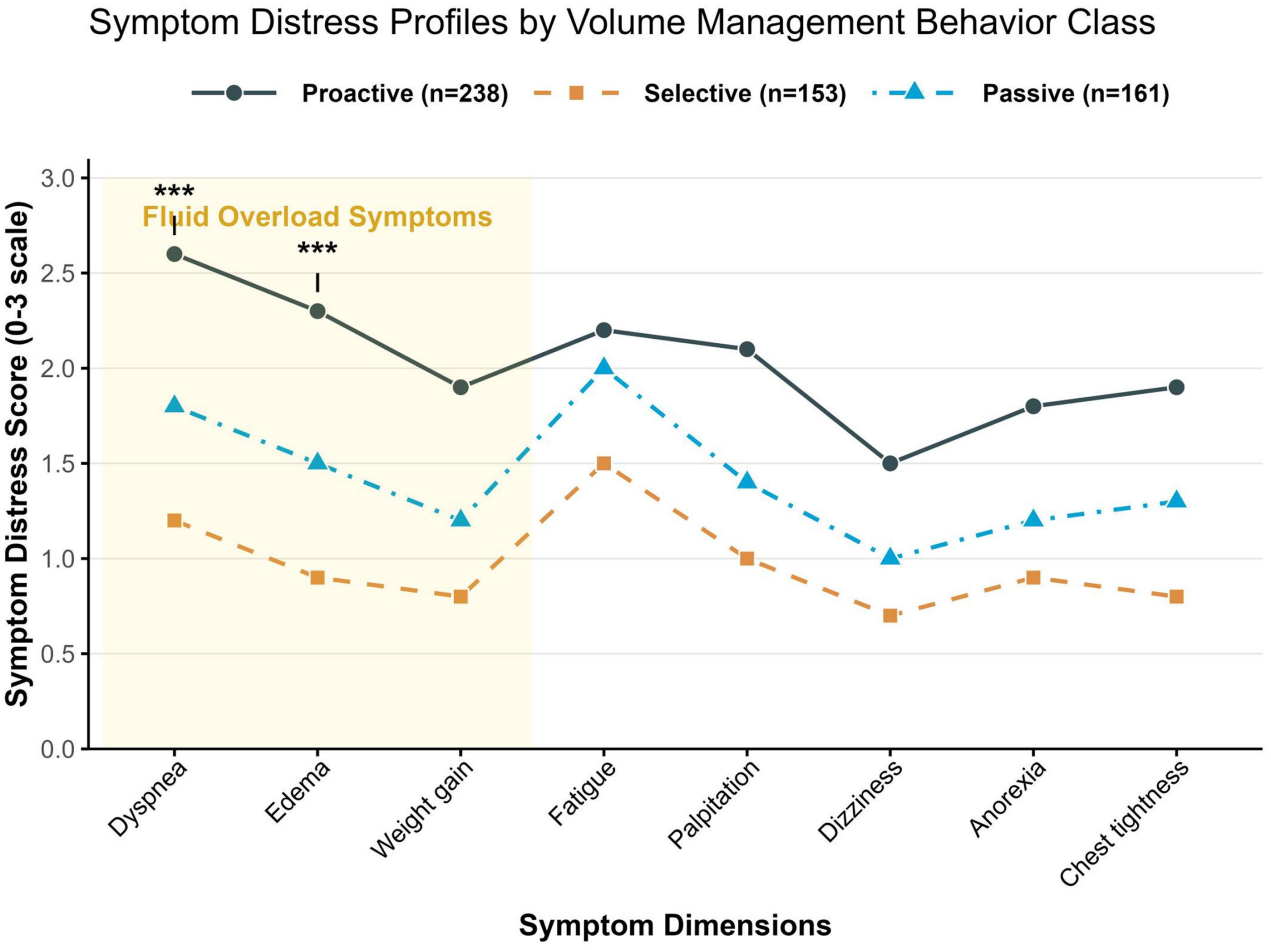
Symptom distress characteristics of patients across different volume management behavior profiles.

Based on the eight symptom items of the SDQ, an analysis of symptom cluster differences was conducted across the three behavioral profiles (Table 4). Scores for all symptoms exhibited a gradient trend: Active Management Type < Selective Adherence Type < Passive Dependence Type (all *P* < 0.001). The degree of variation differed across symptom clusters. Core symptoms of volume overload (dyspnea, edema, and weight gain) showed the most significant between-group differences, accounting for the majority of the total variance in symptom distress (pooled *η*^2^ = 0.47). Among these, dyspnea demonstrated the largest difference (*η*^2^ = 0.51, *F* = 98.32, *P* < 0.001). Neuroendocrine symptoms (fatigue, palpitations) and low-perfusion symptoms (dizziness, loss of appetite, chest tightness) also showed large to very large effect sizes (*η*^2^ = 0.28–0.42). The symptom distress characteristics of patients across different volume management behavioral categories are illustrated in Figure 2.

**Table 4:** Comparison of symptom dimension scores across behavioral profiles.

Symptom	Active management type (*n* = 238) ①	Selective adherence type (*n* = 153) ②	Passive dependence type (*n* = 161) ③	*F*-value	*η* ^2^	*P*-value	Between-group differences (LSD test)
Dyspnea	1.2 ± 0.5	1.8 ± 0.6	2.6 ± 0.3	98.32	0.51	<0.001	① < ② < ③
Edema	0.9 ± 0.4	1.5 ± 0.5	2.3 ± 0.6	85.17	0.47	<0.001	① < ② < ③
Weight Gain	0.8 ± 0.3	1.2 ± 0.4	1.9 ± 0.5	76.44	0.43	<0.001	① < ② < ③
Fatigue	1.5 ± 0.6	2.0 ± 0.5	2.2 ± 0.7	64.33	0.38	<0.001	① < ② < ③
Palpitations	1.0 ± 0.4	1.4 ± 0.5	2.1 ± 0.6	58.21	0.42	<0.001	① < ② < ③
Chest Tightness	0.8 ± 0.3	1.3 ± 0.4	1.9 ± 0.5	41.33	0.32	<0.001	① < ② < ③
Dizziness	0.7 ± 0.3	1.0 ± 0.4	1.5 ± 0.5	32.15	0.18	<0.001	① < ② < ③
Loss of Appetite	0.9 ± 0.4	1.2 ± 0.5	1.8 ± 0.6	28.77	0.16	<0.001	① < ② < ③

Data presented as mean ± standard deviation. Group comparisons were performed using one-way ANOVA, with *post-hoc* pairwise comparisons conducted using LSD test. Homogeneity of variances was confirmed by Levene's test (all *P* > 0.05). *η*^2^: partial eta squared (effect size: small >0.01, medium >0.06, large >0.14).

### 3.5 Independent association analysis between volume management behavior profiles and symptom distress

To clarify the independent predictive effect of latent classes of volume management behaviors on symptom distress while controlling for potential confounding factors, a multiple linear regression model was employed. The total symptom distress score was set as the dependent variable. Behavioral profile classification served as the core independent variable (dummy-coded with the “Active Management Type” as the reference). The following clinical and demographic variables were forced into the model (enter method) as covariates for adjustment: age (continuous), gender (male = 0, female = 1), education level (junior high school and below = 0, high school and above = 1), NYHA class (dummy-coded with Class II as the reference), LVEF (continuous), and number of comorbidities (continuous).

Multiple linear regression analysis revealed that, after controlling for the effects of age, gender, education level, NYHA class, LVEF, and number of comorbidities, the volume management behavior profile was the strongest independent predictor of the total symptom distress score. Compared to patients with the Active Management Type, those with the Passive Dependence Type had a significantly higher symptom distress score by 5.82 points (95% CI: 4.21–7.43, *P* < 0.001), while those with the Selective Adherence Type had a significantly higher score by 2.91 points (95% CI: 1.62–4.20, *P* < 0.001). Additionally, worse NYHA functional class (Class III: *β* = 1.87; Class IV: *β* = 3.15) and lower LVEF (*β* = 0.92) were also independently associated with higher levels of symptom distress. The model explained 41% of the variance in the total symptom distress score (adjusted *R*^2^ = 0.41). Significant variables retained in the final model are presented in Table 5.

**Table 5:** Multiple linear regression analysis of factors associated with symptom distress in CHF patients.

Variable	*β* (95%CI)	*P*-value	Standardized *β*	VIF
Behavior Profile (Ref: Active Management Type)				
Selective Adherence Type	2.91 (1.62–4.20)	<0.001	0.32	1.28
Passive Dependence Type	5.82 (4.21–7.43)	<0.001	0.61	1.35
NYHA Class (Ref: Class II)				
Class III	1.87 (0.75–2.99)	0.001	0.19	1.32
Class IV	3.15 (1.52–4.78)	0.002	0.28	1.41
LVEF (per 10% decrease)	0.92 (0.31–1.53)	0.003	0.15	1.22
Age (per 10-yearincrease)	0.48 (−0.12 to 1.08)	0.115	0.08	1.18
Education Level (High school or above)	−0.67 (−1.45 to 0.11)	0.093	−0.06	1.09
Number of Comorbidities	0.41 (−0.05 to 0.87)	0.081	0.07	1.15
Constant	6.73 (4.25–9.21)	<0.001	–	–

Adjusted *R*^2^ = 0.41; *F*(10,541) = 28.55, *P* < 0.001.

Model diagnostics: All VIFs <2.0; Durbin-Watson = 1.92; residual normality test (Shapiro–Wilk *P* = 0.312).

## 4 Discussion

This study, for the first time using latent profile analysis, identified three distinct volume management behavior patterns among 552 patients with CHF: the Active Management Type (43.1%), the Selective Adherence Type (27.7%), and the Passive Dependence Type (29.2%). This classification framework transcends the traditional simplistic dichotomy of “high vs. low adherence,” revealing the complex heterogeneity in patients' self-management behaviors and providing a critical foundation for implementing precision interventions.

Patients of the Active Management Type employ systematic self-monitoring, such as daily weighing at fixed times to promptly detect abnormal weight changes, and preventive behaviors like proactive sodium restriction to mitigate volume overload risk at its source, effectively maintaining volume balance. This aligns closely with the “ABC model” of heart failure self-management (Avoid fluid overload; Balance electrolytes; Comply with medication) (18). Concurrently, their behavioral pattern fully embodies the concept of “Patient Empowerment” advocated by the Heart Failure Association of Europe (19, 20), which emphasizes equipping patients with the knowledge and ability for self-management, enabling them to become the primary agents of their own health care for better disease control.

The Selective Adherence Type (27.7%) represents a contradictory group characterized by a cognition-behavior disconnect. Their key contradiction lies in having symptom recognition capabilities relatively close to the Active Management Type, allowing them to accurately identify HF-related symptoms. However, this group exhibits significant deficiencies in dietary management and healthcare-seeking behaviors, manifesting as avoidance of dietary control with very low adherence to sodium restriction (often consuming high-sodium foods) and a tendency to delay seeking medical care, opting to postpone action even when obvious symptoms appear. This group demonstrates a typical pattern of medical dependency—they are willing to comply only with explicit medical instructions from doctors, such as taking medications on time, but show clear avoidance towards behaviors requiring autonomous lifestyle changes, like sodium restriction. This phenomenon highly corresponds with Bandura's self-efficacy theory (21), which posits that an individual's belief in their own capabilities influences their behavioral choices and execution.

Patients of the Passive Dependence Type (29.2%) exhibit comprehensive deficiencies in volume management behaviors. This group had significantly more severe disease than the other two types, with the proportion of NYHA class IV reaching 20.5% (compared to only 5.0% in the Active Management Type) and a median NT-proBNP level as high as 12,500 pg/mL (compared to 3,850 pg/mL in the Active Management Type), indicating they are in the advanced stages of HF with severely impaired cardiac function. Theoretically, their behavioral pattern aligns with Leventhal's illness representation theory (22), which suggests that an individual's perception of their illness influences their coping behaviors. Passive Dependence Type patients view heart failure as an “acute episodic event” rather than a chronic condition requiring long-term, daily management, leading them to take action only during acute exacerbations with severe symptoms, lacking daily preventive and management behaviors.

The finding that nearly 30% of patients fall into the Passive Dependence Type reflects the current weakness of self-management support systems for CHF patients in China. On one hand, the long-established high-sodium dietary habits among the Chinese population pose significant cultural and lifestyle barriers for patients attempting sodium restriction, indicating insufficient dietary culture adaptability. On the other hand, primary healthcare has not fully realized its potential in the long-term management of CHF patients, who struggle to obtain timely and effective professional guidance during daily self-management, revealing a notable gap in primary care accessibility. These factors collectively contribute to the high proportion of Passive Dependence Type CHF patients in China (23–25).

This study reveals the distribution characteristics of symptom distress and its association with behavioral patterns. Patients with the passive dependence type exhibited the classic triad of volume overload symptoms (dyspnea: 2.6 ± 0.3; edema: 2.3 ± 0.6; weight gain: 1.9 ± 0.5). The pathophysiological basis for this lies in dysregulation of the RAAS axis and the phenomenon of aldosterone escape (26, 27). Furthermore, within the passive dependence group, the symptoms of fatigue and palpitations formed a neuroendocrine activation symptom cluster. The pathophysiological mechanism behind this cluster is closely linked to excessive RAAS activation. Firstly, RAAS activation induces muscle metabolic disorders, impairing energy supply and leading to fatigue (28). Secondly, RAAS activation stimulates the sympathetic nervous system, resulting in increased heart rate and arrhythmias, which manifest as palpitations (29).

In passive dependence type patients, the occurrence of dizziness (1.5 ± 0.5) and loss of appetite was relatively high. However, these two symptoms are often overlooked in clinical practice, lacking sufficient attention and intervention. The persistent presence of loss of appetite can lead to inadequate nutritional intake, further exacerbating physical weakness and impairing cardiac function recovery and quality of life (30). This indicates that the low-perfusion symptom cluster, although its severity may be relatively lower, holds important early warning value. It serves as a crucial signal indicating disease progression and potential risks, and clinicians should increase their awareness of this cluster. The total symptom distress score for passive dependence type patients reached 16.3 ± 5.2 points, exceeding the readmission threshold set by the Jurgens criteria (31). This signifies an extremely high risk of readmission for this patient type. Nevertheless, few passive dependence type patients have received professional symptom management guidance, highlighting the urgency and necessity for enhanced nursing interventions focused on symptom management in CHF patients. There is a critical need to strengthen patient education and provide specialized guidance on symptom management.

To clarify the influencing factors of symptom distress in CHF patients, this study employed multiple linear regression analysis. The results demonstrated that the volume management behavior profile was the strongest predictor of symptom distress (*β* = 5.82). Its impact on symptom distress far exceeded that of traditional biomedical indicators such as age, gender, and underlying diseases, fully highlighting the critical role of volume management behaviors in symptom control for CHF patients. The *β* value for the passive dependence type reached 5.82, indicating a symptom burden equivalent to that of patients with severely impaired cardiac function (NYHA class IV and LVEF <30%), representing an extremely high level of symptom distress. The active management type, serving as the reference group, exhibited the lowest level of symptom distress, representing the ideal model for volume management. Particularly noteworthy is that after adjusting for various factors including diuretic use, the behavior profile remained the strongest predictor, while the diuretic treatment regimen itself did not demonstrate independent predictive significance. This finding presents an important contrast to existing research: although diuretics are the cornerstone of heart failure treatment (32), this study suggests that a mere medication prescription is insufficient to improve patient outcomes, and behavior patterns may be more important than drug selection. This explains why in clinical practice, some patients still experience poor prognosis even when receiving guideline-recommended diuretic therapy (33).

Based on an in-depth analysis of the relationship between volume management behavior patterns and symptom distress in CHF patients in this study, and considering the characteristics and needs of patients with different behavior types, a typed-graded-stratified intervention strategy is proposed. For passive dependence type patients, who have severe conditions, high symptom distress, and poor self-management capabilities, crisis management should be the top priority (34, 35). Specific measures include: On one hand, provide patients with an electronic weight monitor equipped with a real-time alarm function. This alarm triggers when a patient's weight increases by more than 2 kg within 3 days, alerting both the patient and healthcare providers to changes in volume status for timely intervention. On the other hand, provide low-sodium salt substitutes formulated with potassium chloride replacing 50% of the sodium chloride. This helps patients reduce sodium intake while supplementing potassium to maintain electrolyte balance.

The core issue for selective adherence type patients is the cognition-behavior disconnect; they possess some ability to recognize symptoms but fall short in behavioral execution. Therefore, the intervention focus should be on bridging this gap, employing motivational interviewing-driven interventions. Conduct visual dietary education to intuitively demonstrate the hazards of a high-sodium diet, thereby enhancing patients' awareness of the importance of sodium restriction (36). Although active management type patients have strong self-management abilities, long-term disease management can lead to burnout, potentially affecting the sustainability and effectiveness of their efforts. Thus, the intervention focus should be on preventing burnout and upgrading support. Develop a heart failure management APP (37) that automatically unlocks achievement badges when a patient successfully completes management tasks (e.g., weight monitoring, low-sodium diet, medication adherence) for 7 consecutive days. This reward mechanism aims to stimulate patients' management motivation and maintain their drive. Additionally, consistent with findings from a recent systematic review (38), implement family supporter training to educate patients' families on heart failure knowledge and early symptom recognition, particularly early signs like worsening fatigue. This enables family members to assist patients in daily management, providing more support and encouragement, thereby enhancing the patient's self-efficacy.

The interpretability of these findings should consider certain limitations. The cross-sectional nature of the study means that causal directions cannot be firmly established. Also, the convenience sampling method, though adequate for the LPA, may limit the generalizability of the profile distribution to the broader CHF population. Future longitudinal research is warranted to confirm the stability of these profiles and their predictive value for clinical outcomes. This study's principal contribution is its novel, phenotype-based perspective on self-management. It challenges the simplistic view of adherence by revealing that it is not just the degree but the pattern of behaviors that is critically associated with symptom burden. This paradigm shift is a significant step towards precision behavioral medicine in heart failure care, enabling the move from one-size-fits-all advice to interventions customized to address the specific deficits of each behavioral profile, such as low self-confidence in the selective adherence group or lack of routine monitoring in the passive dependence group.

## 5 Conclusion

This study employed latent profile analysis to categorize 552 CHF patients into three distinct volume management behavior profiles: active management type, selective adherence type, and passive dependence type. It systematically elucidated the intrinsic relationship between different behavioral patterns and symptom distress. The results indicate that passive dependence type patients endure a severe symptom burden, fundamentally due to a vicious cycle formed by behavioral deficiencies such as lack of weight monitoring and high-sodium dietary habits. This study further confirms that volume management behavior is a strong independent predictor of symptom distress, with explanatory power surpassing traditional clinical indicators. This finding emphasizes that in the holistic management of CHF, behavioral interventions should not merely serve as auxiliary measures but should be regarded as a core strategy equally important as pharmacological therapy and device-based interventions. Future efforts should focus on constructing an integrated management model of “behavioral typing–precision intervention–digital support,” leveraging smart technologies to enable early identification of high-risk patients and deliver personalized behavioral guidance. This approach will genuinely improve patients' symptom experience, reduce readmissions, and enhance quality of life and long-term prognosis.

## Data Availability

The original contributions presented in the study are included in the article/Supplementary Material, further inquiries can be directed to the corresponding author.
